# (Neo)adjuvant chemotherapy and interdigitated split-course hyperfractionated radiation in high risk soft tissue sarcoma – Results from a large single-institution series

**DOI:** 10.1038/s41598-019-43794-3

**Published:** 2019-05-13

**Authors:** Riikka Nevala, Erkki Tukiainen, Maija Tarkkanen, Tom Böhling, Carl Blomqvist, Mika Sampo

**Affiliations:** 1 Comprehensive Cancer Center, Helsinki University Hospital (HUH), Helsinki, Finland; 2 University of Helsinki, Helsinki, Finland; 3 Department of Plastic Surgery, HUH, Helsinki, Finland; 4 Department of Pathology, University of Helsinki and HUSLAB, HUH, Helsinki, Finland; 5 Department of Oncology, Orebro University Hospital, Orebro, Sweden

**Keywords:** Sarcoma, Surgical oncology

## Abstract

A single-institution series using a (neo)adjuvant chemotherapy and interdigitated hyperfractionated split-course radiation therapy (CRT) treatment protocol for soft tissue sarcoma was reviewed. Our specific aims were to study recurrence rates and long-term toxicity. Between 1998 and 2016, 89 patients with non-metastatic soft tissue sarcoma were treated with surgery combined with six courses of doxorubicin and ifosfamide and hyperfractionated radiation therapy (42–60 Gy/1.5 Gy twice daily). Patients were considered being at high risk if tumour malignancy grade was high and the tumour fulfilled at least two of the following criteria: size >8 cm, presence of necrosis or vascular invasion. The mean age of the patients was 50.7 years. With a median follow-up of 5.4 years for survivors, the local control rate was 81.4%. Six (7%) patients progressed during neoadjuvant CRT. Seven (8%) patients discontinued the treatment due to toxicity. Eighty-six patients were operated and three (3%) of these developed a long-term complication. The estimated metastasis-free survival was 47.6% and overall survival 53.0% at five years. The limb-salvage rate was 93%. The limb-salvage rate, local control and complication rates were good in these patients with high risk soft tissue sarcoma. Metastases-free survival and overall survival rates were less satisfactory, reflecting the aggressive nature of these tumours.

## Introduction

Treatment of soft tissue sarcoma (STS) aims to ensure adequate local control (LC) without major disability and to prevent distant metastases. Radiation therapy (RT) combined with surgery improves LC and enables less mutilating surgery^1^. However, RT has no documented effect on metastases-free survival (MFS)^1^. Twenty to 30% of STS patients develop metastases and most of them die from the disease^2^.

The role of adjuvant systemic therapy in STS is still controversial. In a meta-analysis from 2008, the combination of doxorubicin and ifosfamide was associated with an overall survival (OS) benefit and an absolute risk reduction of 11%^3^. Many patients with STS at high risk of developing metastatic disease are also at risk of local recurrence (LR). Thus, these patients have indications for both RT and chemotherapy (CT). Delaying RT may expose the patient to an increased risk of LR. Delaying CT may expose the patient to growth of subclinical metastases during RT.

The behavior of high-grade STS can be aggressive. The growth rate of pulmonary metastases from sarcomas can be rapid, up to a volume doubling time of only 7–9 days^4,5^. Conventionally fractionated RT has a duration of 6–7 weeks. Theoretically during that time aggressively proliferating occult metastases can increase their volume 64–128-fold. Similarly, four to six CT cycles may enable local tumour growth of more than 100-fold, if the sarcoma does not respond to CT. Thus, a hyperfractionated split-course RT interdigitated between CT cycles (CRT) offers a theoretically interesting option to avoid these problems.

In the present study, we retrospectively reviewed all STS patients at high risk who received hyperfractionated split-course RT interdigitated between (neo)adjuvant doxorubicin and ifosfamide with special interest on outcome and treatment-related long-term complications.

## Patients and Methods

Eighty-nine high-risk patients were treated for local STS by the Soft Tissue Sarcoma Group at Helsinki University Hospital (HUH) during 1998–2016 with CRT. The study was approved by the HUH Ethics Committee and the Ministry of Social and Health Affairs. All methods were performed in accordance with the relevant guidelines and regulations. In Finland, National Institute for Health and Welfare can issue a permission to use patient data for retrospective studies and therefore informed patient consent was not gathered.

Our treatment protocol for STS was set up in 1987. The treatment plan of all new STS patients is decided by multidisciplinary team (MDT) consisting of oncologists, plastic surgeons, radiologists and pathologists. Staging procedures include magnetic resonance imaging (MRI) or computed tomography or both of the primary tumour and an ultrasound-guided or computed tomography-guided core needle biopsy and fine needle aspiration. A computed tomography of the lungs is recommended for all patients. Surgery with wide margins is preferred when feasible. A wide margin is achieved if a cuff of healthy tissue is at least 2.5 cm or an uninvolved fascia surrounds the whole tumour periphery. RT is recommended if the surgical margin is less than 2.5 cm or if tumour cell contamination is suspected. Preoperative RT is recommended if the tumour is assessed to be inoperable.

**Figure 1:**
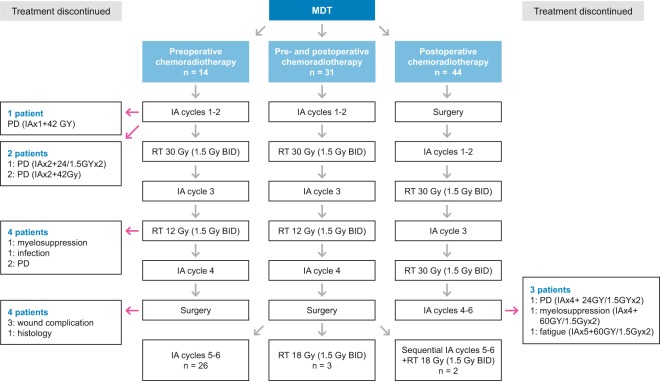
Treatment protocol used by our multidisciplinary team (MDT). BID: twice daily; IA: doxorubicin-ifosfamide; PD: progressive disease; RT: radiation therapy.

Our adjuvant CT treatment protocol was set up in 1998: patients with WHO performance status 0–1 are offered adjuvant CT if the tumour malignancy grade is high (3 in a three-tiered scale) and the tumour fulfills at least two of the following criteria: size >8 cm (in synovial sarcomas >5 cm), presence of necrosis or vascular invasion according to the guidelines of the Scandinavian Sarcoma Group (SSG)^6^. The recommended CT regimen is six cycles of doxorubicin and ifosfamide (IA) with 21-day intervals. Our protocol of interdigitated six CT courses and split-course hyperfractionated RT was derived from the SSG IX for Ewing’s sarcoma^7^. In this protocol, patients with inoperable tumours or with inadequate margins received split-course hyperfractionated RT (1.5 Gy twice daily) in two CT breaks to a total dose of 42–60 Gy (Fig. 1).

Neoadjuvant CT combined with interdigitated split-course hyperfractionated RT is preferred when the tumour is aggressive and growing fast and the margin in definite surgery is likely to be intralesional, marginal or an amputation seemed unavoidable. If the patient needs both CT and RT as adjuvant therapy, interdigitated hyperfractionated therapy is preferred instead of sequential CT and RT treatment. For abdominal or for other tumor localization, where acute toxicity of RT is to be expected, sequential therapy is preferred.

CRT treatment starts with two cycles of CT, which consists of doxorubicin (50 mg/m^2^) and ifosfamide (5 g/m^2^) (IA) combination (q21) (Fig. 1). Granulocyte stimulating factor (G-CSF) is used, if the risk of infection is considered high or if the low white blood cell count is going to cause a delay of CT. After two CT cycles, hyperfractionated RT 30 Gy/1.5 Gy twice a day for ten days with an interfraction interval of at least 6 hours is delivered. After the first course of RT a further hyperfractionated RT 12 Gy/1.5 Gy twice a day is delivered during the interval between CT cycle 3 and 4. Before surgical treatment MRI of primary tumour and computed tomography of lungs are performed to evaluate the treatment response and to exclude disseminated disease.

Limb-sparing surgery is planned whenever feasible. Postoperatively patients receive two cycles of IA depending on the evaluation of histologic response to preoperative treatment in MDT discussion. Patients with positive microscopic margins are re-operated and if this is not feasible or patient refuses of amputative surgery, a RT boost of 18 Gy (1.5 Gy twice daily) is delivered to a reduced target volume. Computed tomography-based treatment planning and individual fixation methods are used in RT. The target volume is defined as the involved muscle compartment in the transversal direction, with a margin of at least 5 cm longitudinally. Common Terminology Criteria for Adverse Events (CTCAE) Version 4.0 is used to report adverse effects.

The equivalent dose of an accelerated hyperfractionated treatment scheme with 1.5-Gy fractions compared to a fractionation scheme with 2-Gy fractions is calculated by the linear quadrate (alfa/beta) formula modified by Thames and Hendry^8^ to account for incomplete repair of sublethal damage when fraction interval is less than 24 hours. The equivalent dose is calculated according to the formula:

$${\rm{TE}}={\rm{N}}\ast {\rm{d}}(\mathrm{alfa}/\mathrm{beta}+{\rm{d}}\ast (1+{\rm{hM}})),$$

where, TE = total effect, N = number of fractions, d = dose per fraction, alfa/beta = measure of the fractionation sensitivity of the tissue, and hM = the correction term for incomplete repair of sublethal damage, dependent of repair half time, interval between fractions and number of fractions per day^9^. An alfa/beta ratio of 10 Gy for acute, and 3 Gy for late effects and a repair half time of 1.2 h for acute and 3.5 h for late effects are used. With these assumptions an accelerated hyperfractionated RT scheme of 1.5 Gy twice daily should be equivalent to a schedule of 2 Gy × 1 for late effects, while the acute effects should be slightly less. Total doses are reduced 15% from 50 Gy to 42 Gy for microscopic and 70 Gy to 60 Gy for macroscopic disease to account for the radiosensitizating effect of doxorubicin and the improved effect of the shorter treatment time when compared to our dose recommendations of RT alone.

Tumour size is defined as the largest diameter of the tumour in the surgical specimen reported by the pathologist, or in the case of neoadjuvant treatment the largest measure in pretreatment MRI/computed tomography. Histological malignancy of the tumour is determined according to the French grading system^10^. After formalin fixation, the surfaces of specimens are painted and dissected. The margins are measured from histological slides. All diagnoses are verified by an experienced sarcoma pathologist.

All patients have a regular follow-up. Patients with high-grade sarcoma undergo a chest X-ray every two months during the first two years, and thereafter three times annually up to five years. Clinical control and a computed tomography or a MRI scan of the primary tumour region are planned six months postoperatively and thereafter once every six months up to two years and thereafter annually up to five years. However, the follow-up of synovial sarcomas is continued up to ten years.

### Statistical methods

LC, MFS, OS and sarcoma-specific survival (SSS) were calculated according to the Kaplan-Meier method. IBM^®^ SPSS^®^ Statistics version 23 (SPSS, Chicago, Illinois, USA) was used for all analyses.

## Results

The present study includes 89 patients with a STS of the trunk wall (n = 9), lower (n = 59) or upper extremity (n = 9), head and neck (n = 1) or deep sites (n = 11) (Table 1). They received treatment with curative intention during 1998–2016. The mean age of the patients at diagnosis was 50.7 years. The three most common histological subtypes were undifferentiated pleomorphic sarcoma, liposarcoma, and synovial sarcoma. Median tumour size was 10.5 cm. All but one tumour were high grade, and all but one were deep-seated. The patient with the liposarcoma of intermediate grade received combination treatment to improve LC because of proximity of major nerves and vessels were expected to compromise surgical margins. In all, preoperative imaging showed that in 21 patients with limb-girdle and limb tumour the definite positive margin would be inevitable with limb-sparing surgery alone. Median follow-up for survivors was 5.4 years.

**Table 1:** Description of tumour, patient and treatment characteristics of 89 patients with interdigitated hyperfractioned radiation therapy and chemotherapy.

Characteristics	No. of patients	%
Sex		
Male	53	60
Female	36	40
Age at Diagnosis (years)		
Mean	50.7	
Range	16.3–75.4	
Referral status		
Virgin	58	65
FNA	9	10
CNB	14	16
Open biopsy	3	3
Intralesional surgery	4	4
Marginal surgery	1	1
Site		
Lower extremity	59	66
Upper extremity	9	10
Trunk	9	10
Head&neck	1	1
Other sites*	11	12
Grade according to French system		
Intermediate	1	1
High	88	99
Depth (trunk wall and extremity tumours)		
Superficial^†^	1	1
Deep^‡^	77	99
Tumour size (cm)^§^		
Median	10.5	
Range	1.5–41.0	
Vascular invasion^¶^		
Present	17	41
Absent	24	59
Necrosis**		
Present	37	86
Absent	6	14
Histological subtype		
UPS	39	44
Liposarcoma	16	18
Synovial sarcoma	11	12
Leiomyosarcoma	8	9
MPNST	4	4
Fibrosarcoma	4	4
Neurofibrosarcoma	1	1
Myxofibrosarcoma	5	6
Epithelioid sarcoma	1	1
Margin (in 86 operated patients)		
Intralesional	16	19
Marginal	59	69
Wide	11	13

Abbreviations: CNB, core needle biopsy; FNA, fine needle aspiration; MPNST, malignant peripheral neural sheath tumour; UPS, undifferentiated pleomorphic sarcoma. *One in thorax cavity, one in vagina, two in prostate and one in retroperitoneum. ^†^Subcutaneous tumours with or without cutaneous extention but without involvement of the deep fascia. ^‡^Tumours with involvement of the deep fascia or deep to it. ^§^Size was not determined in three tumors. ^¶^Vascular invasion in 44 patients with no neoadjuvant treatment, three missing. **Necrosis in 44 patients with no neoadjuvant treatment, one missing.

CRT was given preoperatively in 45 (51%) patients and postoperatively in 44 (49%) patients (Fig. 1). Treatment was stopped in 14 (16%) patients. Reasons for discontinuation were toxicity (7 patients, including myelotoxicity despite G-CSF use in two patients; fatigue in one patient; delayed wound healing in three patients and infection in one patient), progression during treatment (6 patients), and re-classification of tumour histology and grade (one patient). Postoperative CT was omitted from six (7%) patients because of poor histological response to neoadjuvant treatment.

Sixty-six patients were planned to receive 42 Gy/1.5 Gy twice daily and 23 patients were planned to receive 60 Gy/1.5 Gy twice daily. Sixty-three (95%) of 66 patients were treated to 42 Gy as planned whereas 21 (91%) of 23 were treated to 60 Gy as planned. Two patients received only 24 Gy due to progression. One patient planned to receive 42 Gy and two patients planned to receive 60 Gy had minor dose modifications due to acute skin reactions. Three patients received a boost with conventional fractionation.

Sixty (70%) patients received all six courses of IA. Six (7%) patients had dose reductions due to myelotoxicity. G-CSF was used in 57 (66%) patients and 22 (26%) patients received G-CSF after each IA course. Twenty-two (25%) patients were hospitalized because of neutropenic fever and one (1%) patient because of influenza A without neutropenia. Other reasons of IA dose or cycle modification were wound complication (two patients, 2%), central nervous toxicity from ifosfamide (one patient, 1%), oesophageal mucositis (one patient, 1%), deteriorated performance status (one patient, 1%) and re-classification of tumour grade and histology (one patient, 1%).

Three (3%) patients could not be operated due to progression. Definite margin was intralesional, marginal and wide in 16 (19%), 59 (69%) and 11 (12%) out of 86 operated patients, respectively. Seven patients with limb and limb-girdle tumour refused of amputation and had a definite microscopically positive margin whereas nine patients out of 21 patients preoperatively evaluated to have definite positive margin with limb-sparing surgery alone had limb-sparing surgery with negative margin after neoadjuvant treatment. Sixty-two (72%) patients were operated with direct closure. Nine (10%) patients required a microvascular flap and eleven (13%) a pedicled flap. Vascular reconstruction was performed on four (5%) patients. Reoperation was necessary in eight (19%) patients out of 42 operated patients after neoadjuvant treatment due to wound necrosis (four patients), haemorrage (two patients), and infection (two patients). Reoperation was necessary in four (9%) patients out of 44 patients with adjuvant CRT due to wound necrosis (two patients), and infection (two patients). Three patients received a microvascular flap as part of reconstruction for complications.

**Figure 2:**
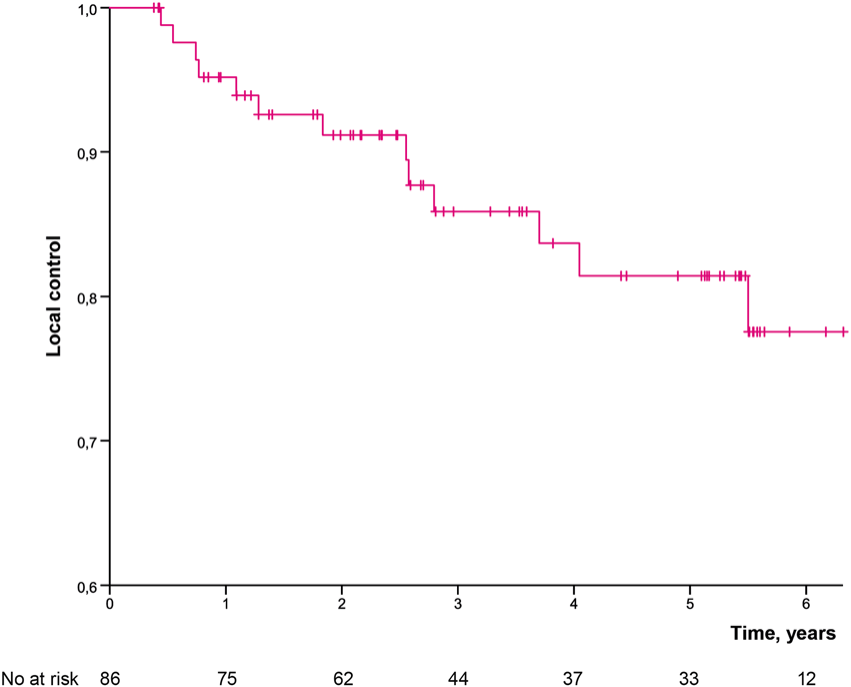
Local control by time in 86 operated patients.

**Figure 3:**
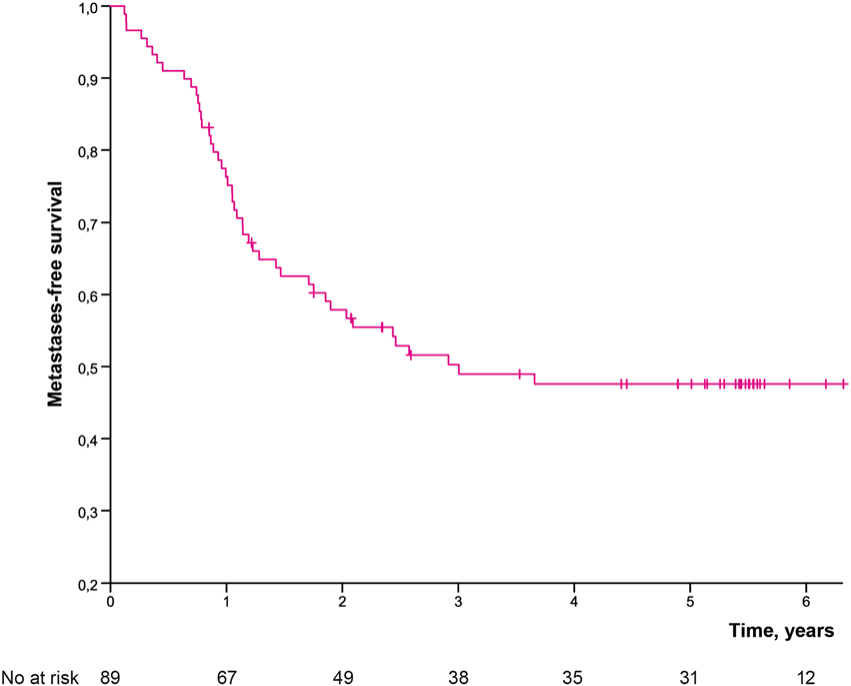
Metastases-free survival by time for the whole population.

**Figure 4:**
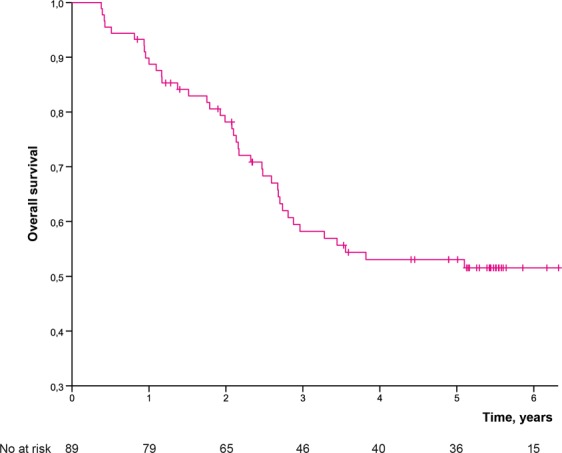
Overall survival by time for the whole population.

Five (7%) out of 68 patients with tumour of extremity or limb girdle had an amputation (one hemipelvectomy, two rotationplasties, two amputations) yielding a limb-salvage rate of 93%. Fourteen (16%) out of 86 operated patients developed LR yielding estimated LC of 81.4% at five years (Fig. 2). Two patients had a late LR at 5.5 years and at 12 years. The estimated MFS was 50.3% at three years, and 47.6% at five years (Fig. 3). SSS was 60.1% at three years and 56.2% at five years. OS was 58.2% at three years and 53.0% at five years (Fig. 4) for the whole study population.

Three patients (3%) developed moderate or severe long-term treatment-related toxicity. One (1%) patient had stiffness and severely limited flexion of the knee joint (Grade 3). One (1%) patient suffered from a chronic pain syndrome with allodynia (Grade 3) and one (1%) patient developed late rupture of the wound eight months after finishing treatment (Grade 3). No treatment-related deaths were recorded.

## Discussion

In the present retrospective study 89 STS patients with high risk of developing both local recurrence and metastatic disease had LC rate of 81% and limb-salvage rate of 93% at 5 years after surgery and (neo)adjuvant CRT. The patients were highly selected, representing only 7% of the STS patients treated by our group during the same period.

Disease-progression in six of our patients during (neo)adjuvant therapy underscores the fast growth rate of high-grade STS. The long-term survival remained also unsatisfactory with approximately 50% of patients dying from their disease despite the aggressive therapy. However, this can partly be anticipated as adjuvant CT has only a moderate effect on survival^3^.

The first neoadjuvant CRT protocol of STS was published by Eilber and colleagues in 1980^11^. Patients were treated with preoperative intra-arterial doxorubicin and hypofractionated RT. An impressive LC rate of 97% and limb-salvage rate of 92% were reported^11^. However, significant morbidity was seen from intra-arterial doxorubicin used as chemosensitizer^11–19^. This included arterial thrombosis^13,14,16,17^, pain^13,18^, severe local skin reactions^15^ and catheter site infections^13^. Due to local complications intra-arterial infusion was later replaced by intravenous administration of CT^13,14,16,17^.

A summary of the most important efficacy endpoints and toxicity in published studies of CRT in STS is shown in Table 2. Since the aim of most CRT protocols in sarcomas has been the achievement of a high LC without amputation, mostly patients with extremity or limb girdle tumours have been included. In the present study, nine patients out of 21 patients preoperatively evaluated to have definite positive margin with limb-sparing surgery alone had limb-sparing surgery with negative margin after neoadjuvant treatment. LC after CRT and surgery of extremity sarcomas has been good to excellent ranging from 71% to 100% with a variety of RT fractionation and CT regimens^11–38^. In a study by Gronchi *et al*. (2014) preoperative CRT has been used also for localized retroperitoneal sarcoma with LC rate 61% at five years, which has to be considered good for sarcomas at this site^39^. Long-term survival varies significantly among CRT studies (Table 2). Most patient series are small with variable chemotherapeutic agents and RT fractionations and patient characteristics vary significantly. Thus the comparison between the survival rate in our study and other published patient series is difficult, and no firm conclusions can be made on the effect of CRT on overall outcome.

**Table 2:** Studies on chemoradiation therapy for STS reporting survival rates and complications.

Study	Study period	n	CT	RT (Gy)	CRT timing pre/post	CRT C/S	High grade tumors (%)	LC% (at years of FU)	DFS% (at years of FU)	OS% (at years of FU)	Major complications (%)*
Eilber *et al*.^11^	1972–1979	65	A 30 mg × 1 i.a. 3 days	35/3.5	pre	S	80	97 (2.5)	NA	75 (5)	28
Goodnight *et al*.^12^	1980–1984	17	A 25–30 mg i.a. 3 days	35/3.5 or 40/2	pre	S	76	100 (2.5)	59 (2.5)	82 (2.5)	35
Levine *et al*.^13^	1978–1991	55	A 10 mg/m^2^ i.a. 10 days	25/2.5	pre	C	58	85 (5)	51 (5)	69 (5)	25
Mason *et al*.^14^	1983–1985	13	A 10–30 mg i.a. 3 days	40–65/2	pre	NA	85	77 (4.5)	NA	NA	69
Nijhuis *et al*.^15^	1983–1987	11	A 20 mg/m^2^ i.a. 3 days	35/3.5	pre	S	100	100 (7)	55 (7)	55 (7)	45
Mack *et al*.^16^	1984–1996	75	A 30 mg × 1 i.a. 3 days	30/3	pre	S	48	95 (5)	NA	63 (5)	8
Temple *et al*.^17^	1984–1994	40	A 30 mg × 1 i.a. 3 days	30/3	pre	S	NA	97 (5)	NA	79 (5)	13
Temple *et al*.^18^	1986–2002	44	A 30 mg × 1 3 days	30/3 or 33/1.65 × 2	pre	S	45	96 (5)	68 (5)	73 (5)	11
Dincbas *et al*.^27^	1989–2007	44	IA × 6	35/3.5 or 46–50/2	pre	S	NA	82 (5)	47 (5)	70 (5)	30
Mantravadi *et al*.^19^	NA	32	A 10 mg/m^2^ i.a. 10 days	25/2.5	pre	C	100	97 (3)	57 (3)	70 (3)	NA
Brodowicz *et al*.^31^	1992–NA	31	IFADIC × 4 + IFDIC × 2	51/1.7 × 2	post	NA	81	94 (3.5)	77 (3.5)	97 (3.5)	10
Aguiar Jr *et al*.^35^	1995–2004	49	A 20 mg/m^2^ × 3	30/2.5	pre	C	59	82 (5)	47 (5)	58 (5)	42
Greto *et al*.^21^	1998–2011	32	IE × 2	50/2	pre	C	100	NA	53 (4.9)	NA	NA
Mahmoud *et al*.^16^	1999–2012	49	IA × 4–6	63/1.8–2	post	S	92	71 (5)	43 (5)	67 (5)	10
Gronchi *et al*.^22^	2002–2007	135	IE × 3–5	50	pre	NA	100	96 (5)	67 (5)	70 (5)	NA
Gronchi *et al*.^39^	2003–2010	83	I 14 g/m^2^ × 3	50.4/1.8	pre	C	19	63 (5)	44 (5)	59 (5)	22
Kraybill *et al*.^33^	1997–2000	64	MAID × 6	44	pre	S	80	78 (5)	56 (5)	71 (5)	13
Edmonson *et al*.^29^	1994–1997	39	IMAP* × 2 + MAP × 3	45/1.8	pre	C	95	90 (5)	75 (5)	80 (5)	5
Brands *et al*.^37^	1997–2004	27	IA × 4	50.4/1.8–2	post	S	74	85 (5)	66 (5)	80 (5)	0
Nesseler *et al*.^34^	1990–2012	29	A × 6 or MAID × 6	50–56/2	post	C or S	69	96 (5)	58 (5)	72 (5)	21
Stubbe *et al*.^30^	2000–2011	53	IA × 2	60/1.5 × 2 or 50.4–60/1.8–2	pre	C	55	90 (5)	NA	83 (5)	21
MacDermed *et al*.^25^	1995–2008	34	I 2.5 g/m^2^ 5 days	28/3.5	pre	C	94	89 (5)	53 (5)	42 (5)	18
Ryan *et al*.^28^	2002–2005	25	IE × 5 + I × 1	28/3.5	pre	C	88	88 (2)	62 (2)	84 (2)	24
Mullen *et al*.^32^	1989–1999	48	MAID × 6	44/2	pre	S	49	90 (7)	65 (10)	84 (5), 66 (10)	46
Jebsen *et al*.^20^	1998–2007	76	IA × 6	36–45/1.8 × 2	post	S	100	71–90 (5)	NA	NA	2
Lehane *et al*.^26^	1995–2012	29	A 30 mg × 1 3 days	30/1.5 × 2	pre	S	69	88 (5)	NA	87 (5)	14
Okuno *et al*.^23^	2001–2006	38	IMAP × 2 + MAP × 2	45–50/1.8	pre	C	100	NA	69 (3)	82 (3)	NA
Raval *et al*.^24^	1997–2010	16	MAID × 6	44/2	pre	S	100	100 (3)	63 (3)	73 (3)	0
Schliemann *et al*.^38^	1997–2014	104	IA × 4	50.4/1.8–2	post	C	84	89 (5)	68 (5)	76 (5)	0
Present study	1998–2016	89	IA × 6	42–60/1.5 × 2	pre or post	S	99	81 (5)	48 (5)	53 (5)	3

Abbreviations: A, doxorubicin; C, concomitant (RT and CT in same day); CRT, chemoradiotherapy; CT, chemotherapy; DFS, disease-free survival; FU, follow-up; I, ifosfamide; i.a.; intra-arterial; IA, ifosfamide + doxorubicin; IE, ifosfamide + epirubicin; IFADIC, ifosfamide + doxorubicin + dacarbazine; IFDIC, ifosfamide + dacarbazine; IMAP, ifosfamide + mitomycin + doxorubicin + cisplatin; LC, local control; MAID, doxorubicin + ifosfamide + dacarbazine; MAP, mitomycin + doxorubicin + cisplatin; NA, not available; OS, overall survival; RT, radiation therapy; S, sequential (RT and CT in different days). *Toxicity is a sum of fatal complications, complications requiring reoperation or embolectomy and late complications affecting the quality of life.

Complication rates in hyperfractionated RT regimens have been variable. In our study, 19% of patients in neoadjuvant and 9% of adjuvant CRT group needed a re-operation. In other neoadjuvant studies using hyperfractionated low fraction doses, wound complication rates varied between 6% and 14%^26,30^. In two postoperative hyperfractionation studies, no problems of wound healing were reported^20,31^. RT fraction size and preoperative treatment are probably important factors for the risk of developing local complications. In five neoadjuvant studies using hypofractionation (single dose >3 Gy) wound complications requiring intervention were frequent (11–29%)^12,15,25,27,28^. On the other hand, in three neoadjuvant trials with smaller than conventional RT fraction size (<2 Gy) wound complications were very rare^23,29,39^. Also the development of surgical techniques together with lower RT doses per fraction decrease the risk of local problems in recent series.

Despite frequent use of G-CSF the risk of acute CT related toxicity was still relatively high as 26% of our patients were hospitalized due to infections. The Scandinavian Sarcoma Group CRT regimen was associated with a similar risk of acute complications, because one third of patients were hospitalized, received a blood transfusion or experienced fever^20^. Treatment compliance was excellent in the Scandinavian study because 92% of patients completed all six IA courses. Other CT regimens have been considerably more toxic. For example, 97% of patients receiving the MAID regimen experienced grade three or higher toxicities, although these were mostly acute and transitory^33^. Only 59–83% of patients could receive all six chemotherapy cycles^32,33^.

Long-term toxicity was rare (3%) in our series but may have been underestimated due to the short survival of many patients. In other CRT studies, bone fractures (3–7%)^11,30,32^, chronic pain disorders (2–15%)^32,33^, decreased joint movement (3%)^33^, significant motion limitations (6%)^32^ and late sequelae (2%)^20^ have been described. A few treatment related deaths due to secondary myelodysplasia^33^, acute myelogenous leukemias^32^, severe nephrotoxicity^20^ and hypokinetic heart failure^34^ has been reported. No treatment-related deaths occurred in our study.

Hyperfractionated RT interdigitated between CT cycles has been used in only five studies in addition to the present^18,20,26,30,31^, while most other CRT protocols have used once daily fractionation. A recent Scandinavian prospective study had a similar design to our protocol using sequential IA cycles with hyperfractionated RT^20^. The outcomes were similar or better with five-year LR, MFS and OS rates of 12%, 59% and 68%, respectively^20^.

Experience of interdigitated doxorubicin-based CT and hyperfractionated RT in Ewing’s sarcoma has also verified the feasibility of this approach^7,40^. Patients were randomized into conventional RT fractionation with a break for CT or hyperfractionated RT simultaneously with the CT^40^. Five-year OS was similar with hyperfractionated and conventional fractionationated RT (63% and 65%) but LC was slightly, but not significantly better with the hyperfractionated RT. Radiation-related long-term complications were rare without difference between the two arms^40^. A reliable estimation of the efficacy and toxicity of hyperfractionated RT and interdigitated CT compared to sequential treatment in non-Ewing STS would require a similar randomized study. Comparison between the results in Ewing’s sarcoma and our results should be made with caution because patients with Ewing’s sarcoma were significantly younger (25 years or less) than patients in our study (mean age 50.7 years). Furthermore, Ewing’s sarcoma is more sensitive to both radiation therapy and chemotherapy than sarcoma of other histology.

In summary, our protocol with interdigitated CT courses and hyperfractionated split-course RT yielded a satisfactory local control and low long-term complication rate. Our results and previous studies indicate that interdigitated hyperfractionated RT and CT is a feasible method of delivering both treatment modalities in patients with highly aggressive STS, where treatment delays may be detrimental.

## References

[1] Yang JC (1998). Randomized prospective study of the benefit of adjuvant radiation therapy in the treatment of soft tissue sarcomas of the extremity. J. Clin. Oncol..

[2] Gronchi A (2011). Primary extremity soft tissue sarcomas: outcome improvement over time at a single institution. Ann. Oncol..

[3] Pervaiz N (2008). A systematic meta-analysis of randomized controlled trials of adjuvant chemotherapy for localized resectable soft-tissue sarcoma. Cancer.

[4] Blomqvist C, Wiklund T, Tarkkanen M, Elomaa I, Virolainen M (1993). Measurement of growth rate of lung metastases in 21 patients with bone or soft-tissue sarcoma. Br. J. Cancer.

[5] Nakamura T (2017). Impact of tumor volume doubling time on post-metastatic survival in bone or soft-tissue sarcoma patients treated with metastasectomy and/or radiofrequency ablation of the lung. Onco. Targets Ther..

[6] Gustafson P (2003). Prognostic information in soft tissue sarcoma using tumour size, vascular invasion and microscopic tumour necrosis-the SIN-system. Eur. J. Cancer.

[7] Elomaa I (2000). Five-year results in Ewing’s sarcoma. The Scandinavian Sarcoma Group experience with the SSG IX protocol. Eur. J. Cancer.

[8] *Fractionation in Radiotherapy* (eds Thames, H. & Hendry J.) (Taylor and Francis, 1987).

[9] Turesson I, Notter G (1988). Accelerated versus conventional fractionation. The degree of incomplete repair in human skin with a four-hour-fraction interval studied after postmastectomy irradiation. Acta Oncol..

[10] Coindre JM (2006). Grading of soft tissue sarcomas: review and update. Arch. Pathol. Lab. Med..

[11] Eilber FR, Mirra JJ, Grant TT, Weisenburger T, Morton DL (1980). Is amputation necessary for sarcomas? A seven-year experience with limb salvage. Ann. Surg..

[12] Goodnight JE, Bargar WL, Voegeli T, Blaisdell FW (1985). Limb-sparing surgery for extremity sarcomas after preoperative intraarterial doxorubicin and radiation therapy. Am. J. Surg..

[13] Levine EA, Trippon M, Das Gupta TK (1993). Preoperative multimodality treatment for soft tissue sarcomas. Cancer.

[14] Mason M, Robinson M, Harmer C, Westbury G (1992). Intra-arterial adriamycin, conventionally fractionated radiotherapy and conservative surgery for soft tissue sarcomas. Clin. Oncol..

[15] Nijhuis PH (1999). Long-term results of preoperative intra-arterial doxorubicin combined with neoadjuvant radiotherapy, followed by extensive surgical resection for locally advanced soft tissue sarcomas of the extremities. Radiother. Oncol..

[16] Mack LA (2005). Preoperative chemoradiotherapy (modified Eilber protocol) provides maximum local control and minimal morbidity in patients with soft tissue sarcoma. Ann. Surg. Oncol..

[17] Temple WJ (1997). Prospective cohort study of neoadjuvant treatment in conservative surgery of soft tissue sarcomas. Ann. Surg. Oncol..

[18] Temple CL (2007). Preoperative chemoradiation and flap reconstruction provide high local control and low wound complication rates for patients undergoing limb salvage surgery for upper extremity tumors. J. Surg. Oncol..

[19] Mantravadi RV, Trippon MJ, Patel MK, Walker MJ, Das Gupta TK (1984). Limb salvage in extremity soft-tissue sarcoma: combined modality therapy. Radiology.

[20] Jebsen NL (2011). Five-year results from a Scandinavian sarcoma group study (SSG XIII) of adjuvant chemotherapy combined with accelerated radiotherapy in high-risk soft tissue sarcoma of extremities and trunk wall. Int. J. Radiat. Oncol. Biol. Phys..

[21] Greto D (2014). Neoadjuvant treatment of soft tissue sarcoma. Radiol. Med..

[22] Gronchi A (2012). Short, full-dose adjuvant chemotherapy in high-risk adult soft tissue sarcomas: a randomized clinical trial from the Italian Sarcoma Group and the Spanish Sarcoma Group. J. Clin. Oncol..

[23] Okuno S (2016). Chemotherapy, Irradiation, and Surgery for Function-preserving Curative Therapy of Primary Extremity Soft Tissue Sarcomas: Initial Treatment With I-MAP and Inhalation GM-CSF During Preoperative Irradiation and Postoperatively. Am. J. Clin. Oncol..

[24] Raval RR (2017). Evaluating the Role of Interdigitated Neoadjuvant Chemotherapy and Radiation in the Management of High-Grade Soft-Tissue Sarcoma: The Johns Hopkins Experience. Am. J. Clin. Oncol..

[25] MacDermed DM (2010). Primary tumor necrosis predicts distant control in locally advanced soft-tissue sarcomas after preoperative concurrent chemoradiotherapy. Int. J. Radiat. Oncol. Biol. Phys..

[26] Lehane C (2016). Neoadjuvant chemoradiation (modified Eilber protocol) versus adjuvant radiotherapy in the treatment of extremity soft tissue sarcoma. J. Med. Imaging Radiat. Oncol..

[27] Dincbas FO (2014). Neoadjuvant treatment with preoperative radiotherapy for extremity soft tissue sarcomas: long-term results from a single institution in Turkey. Asian Pac. J. Cancer Prev..

[28] Ryan CW (2008). Histologic response of dose-intense chemotherapy with preoperative hypofractionated radiotherapy for patients with high-risk soft tissue sarcomas. Cancer.

[29] Edmonson JH (2002). Chemotherapy, irradiation, and surgery for function-preserving therapy of primary extremity soft tissue sarcomas: initial treatment with ifosfamide, mitomycin, doxorubicin, and cisplatin plus granulocyte macrophage-colony-stimulating factor. Cancer.

[30] Stubbe F (2016). Effective local control of advanced soft tissue sarcoma with neoadjuvant chemoradiotherapy and surgery: A single institutional experience. Cancer Radiother..

[31] Brodowicz T (2000). Intensified Adjuvant IFADIC Chemotherapy for Adult Soft Tissue Sarcoma: A Prospective Randomized Feasibility Trial. Sarcoma.

[32] Mullen JT (2012). Long-term follow-up of patients treated with neoadjuvant chemotherapy and radiotherapy for large, extremity soft tissue sarcomas. Cancer.

[33] Kraybill WG (2010). Long-term results of a phase 2 study of neoadjuvant chemotherapy and radiotherapy in the management of high-risk, high-grade, soft tissue sarcomas of the extremities and body wall: Radiation Therapy Oncology Group Trial 9514. Cancer.

[34] Nesseler JP (2017). A retrospective cohort study to assess adjuvant concurrent chemoradiation (CCRT) compared to adjuvant radiation therapy (RT) in the treatment of grade 2 and 3 extremity soft tissue sarcomas. Radiother. Oncol..

[35] Aguiar Junior S (2009). Neoadjuvant chemoradiation therapy for soft tissue sarcomas of the extremities. Clinics (Sao Paulo).

[36] Mahmoud O (2016). The Impact of Perioperative Chemotherapy Timing in Conjunction With Postoperative External-Beam Radiation Therapy on Extremity Soft-Tissue Sarcomas Outcome. Am. J. Clin. Oncol..

[37] Brandts CH (2012). Adjuvant therapy for resectable high-risk soft tissue sarcoma: feasibility and efficacy of a sandwich chemoradiotherapy strategy. Cancer Chemother. Pharmacol..

[38] Schliemann C (2018). Adjuvant chemotherapy-Radiotherapy-Chemotherapy sandwich protocol in resectable soft tissue sarcoma: An updated single-center analysis of 104 cases. PLoS One.

[39] Gronchi A (2014). Preoperative chemo-radiation therapy for localised retroperitoneal sarcoma: a phase I-II study from the Italian Sarcoma Group. Eur. J. Cancer.

[40] Dunst J (1995). Radiation therapy in Ewing’s sarcoma: an update of the CESS 86 trial. Int. J. Radiat. Oncol. Biol. Phys..

